# A Eugenic Problem

**Published:** 1919-04-26

**Authors:** 


### A Eugenic Problem.

Hitherto eugenists have concerned themselves almost solely with the hygienic eligibility of those about to marry; but what is the eugenist's position with regard to those which have developed disease or whose kin have developed disease after matrimony? What is he to advise when a wife develops consumption or chorea? What is he to advise if several blood relations of the husband evince signs of insanity?

These questions are suggested by a case which has recently appeared in the paper where the husband wished to separate from his wife on account of the disease, consumption, from which he was suffering, and the judge who tried the case ordered restitution of conjugal rights. A consumptive husband is liable to infect his wife; and he is liable to procreate unhealthy progeny, and from this point of view of health and of eugenics separation will be desirable. But from the point of view of law separation is not permissible, and from the point of view of love almost impossible. And even where separation is permissible by law, and possible for love, who is to interrupt or terminate matrimonial relationship? Is the man or his wife to decide? Is the healthy or unhealthy partner to adjudge the matter? Or is the decision to be in the hands of a doctor? The question is not easy in a case of that kind; and it is still more difficult if it be a case of disease appearing in kin. We know a case where two brothers and a sister of a husband became insane after the husband's marriage; two children have since been born, and it is difficult to see who could have intervened to prevent it.

The moral would almost seem to be that no interference in the interests of eugenics by a third party is likely to be very effective. In spite of injury to the race and expenses to the community, the responsibility of decision in matrimonial matters must be left mainly in the hands of those married or about to marry; and the best eugenic measure in most cases is not law but education, and not coercion but public opinion.

